# m^6^A-mRNA Methylation Regulates Gene Expression and Programmable m^6^A Modification of Cellular RNAs With CRISPR-Cas13b in Renal Cell Carcinoma

**DOI:** 10.3389/fgene.2021.795611

**Published:** 2022-01-21

**Authors:** Ying Gan, Aolin Li, Jun Liu, Xiaofei Wang, Zhenan Zhang, Qinhan Li, Xiongjun Ye, Lin Yao, Qian Zhang

**Affiliations:** ^1^ Department of Urology, Peking University First Hospital, Beijing, China; ^2^ Beijing Key Laboratory of Urogenital Diseases (male) Molecular Diagnosis and Treatment Center, Beijing, China; ^3^ Urology and Lithotripsy Center, Peking University People’s Hospital, Peking University, Beijing, China; ^4^ Peking University Applied Lithotripsy Institute, Peking University, Beijing, China

**Keywords:** m6A, gene expression, Programmable Modification, CRISPR-Cas13b, Renal Cell Carcinoma

## Abstract

**Background:** N^6^-methyladenosine (m^6^A) is the most extensive messenger RNA modification. Despite recent advances in the biological roles of m^6^A, its role in the development and progression of renal cell carcinoma (RCC) remains unclear.

**Methods:** In this study, we gained the transcriptome-wide m^6^A profile and gene expression pattern in RCC and paired adjacent peritumoral tissues by meRIP-seq and RNA-seq. m^6^A modifications of mRNAs were validated by meRIP-qPCR in tissues, and targeted methylation or demethylation was validated by using a CRISPR-Cas13b-based tool in RCC cell lines.

**Results:** Our findings showed that there were 13,805 m^6^A peaks among 5,568 coding gene transcripts (mRNAs) in adjacent tissues and 24,730 m^6^A peaks among 6,866 mRNAs in tumor tissues. Furthermore, m^6^A modification sites were usually located in the coding sequences (CDS), and some near the start and stop codons. Gene Ontology analysis revealed that coding genes had differential N^6^-methyladenosine sites and were enriched in kidney development and cancer-related signaling pathways. We also found that different levels of m^6^A modifications could regulate gene expression.

**Conclusion:** In summary, our results provided evidence for studying the potential function of RNA m^6^A modification and m^6^A-mediated gene expression regulation in human RCC.

## Introduction

Renal cell carcinoma (RCC) is one of the most common malignancies in the genitourinary system. Kidney cancer is the sixth most common cancer in men, with 73,820 estimated new cases and 14,770 estimated deaths a year in the United States, according to the latest cancer data (Siegel et al., 2019). Clear cell renal cell carcinoma (ccRCC) is the most common type of renal cell carcinoma, accounting for 80%. In clinical practice, 16% of ccRCCs were diagnosed with metastasis at the initial time, with a 5-year survival rate of 12% (Rydzanicz et al., 2013). Although the oncology research and surgical treatment of RCC have developed rapidly, the prognosis of RCC has not improved significantly. For RCC *in situ*, 20%–30% of patients relapse after initial surgical treatment, and no treatment has been shown to reduce tumor recurrence and improve prognosis (Patel et al., 2016). In recent years, targeted agents have been shown to prolong survival and prognosis in patients with metastasis, but the median survival is still less than 3 years. Besides, in clinical practice, drug resistance and economic burden are still two prominent problems. Therefore, the study on the pathological mechanism and new therapeutic targets of RCC is still a challenging exploration.

The role of RNA in a variety of cellular processes has attracted a lot of attention and has become a rapidly developing field in the past decade. More than 100 chemically modified nucleotides have been found in different types of RNAs, such as mRNA, tRNA, rRNA, snRNA, etc. The modified RNA plays a crucial role in the post-transcriptional regulation of gene expression. In eukaryotes, m^6^A is the most common form of mRNA modification, whose abundance accounts for 0.1%–0.4% of the total adenosine residues (Meyer et al., 2012; Zhu H et al., 2019). In general, m^6^A is highly conserved between humans and mice, located in the 3′ terminal non-coding region, near the stop codon and long internal exons, and is closely associated with altered RNA stability, intracellular distribution, splicing, and translation (Csepany et al., 1990; Dominissini et al., 2012; Meyer et al., 2012). The cellular m^6^A state is regulated by a set of genes called “writers” (METTL3, METTLL4, and WTAP), “erasers” (FTO and ALKBH5), and “readers” (YTHDF2, YTHDF2, YTHDF3, YTHDCL, and YTHDC2) (Fustin et al., 2013; Batista et al., 2014; Lin et al., 2016; Zhang et al., 2016; Bertero et al., 2018). The writer forms a multisubunit methyltransferase complex that could upregulate m^6^A levels, while the eraser could downregulate m^6^A levels, making this event reversible (Meyer et al., 2012; Dominissini et al., 2012).

In addition, it was demonstrated that METTL3, a major RNA N^6^-adenosine methyltransferase, could promote hepatocellular carcinoma progression through YTHDF2-dependent post-transcriptional silencing of SOCS2. Although the m^6^A modification of RNA has been reported to be associated with the occurrence of different types of cancer, the relationship between m^6^A-related genes and RCC remains unclear (Aguilo et al., 2015; Shah et al., 2017; He et al., 2018; Panneerdoss et al., 2018; Zhu W et al., 2019; Fukumoto et al., 2019; Zhang et al., 2019). In this study, we used m^6^A-RIP-seq and RNA-seq to research the m^6^A modification profile and mRNA expression profile in RCC. In addition, we performed CRISPR-dCas13b fusion proteins to target methylation or demethylation of differentially methylated mRNAs (Wilson et al., 2020; Li et al., 2020a). This proved that abnormal RNA m^6^A modifications could directly modulate gene expressions in RCC. Finally, we hope this study would facilitate further investigations of potential functions of m^6^A modification in RCC pathogenesis.

## Materials and Methods

### Patients and Samples

Five pairs of primary renal cell carcinoma (RCC) samples and adjacent peritumoral tissues with informed consent of the patients were gained in the Urology Department of Peking University First Hospital (PKUFH), Beijing, China. This study followed the Helsinki declaration and was approved by the Institutional Ethical Review Board of PKUFH. Samples were obtained immediately after surgical resection and stored in liquid nitrogen after rapid freezing for the subsequent RNA isolation.

### RNA m^6^A and mRNA Sequencing

MeRIP-seq and RNA-seq were performed by Cloudseq Biotech, Inc. (Shanghai, China) as described previously (Dominissini et al., 2013). Briefly, total RNAs were extracted from five pairs of tumor and adjacent peritumoral tissues using TRIzol (Invitrogen). Next, total RNAs were broken into almost 100 nt fragments and were incubated with anti-m^6^A antibody (Manga) for 2 h at 4°C. The beads were prepared and incubated with the total RNAs for 2 h at 4°C. Then the mixture was washed, and the m^6^A-bound RNA was purified with TE buffer. After purification, the library was constructed by Prep Kit (Illumina) on the Hiseq 3000.

### Sequencing Data Analysis

After obtaining the sequencing data of control and IP samples, the read segment data should be preprocessed (such as filtering the read segment with poor sequencing quality), and then all the read segment sequence mapping of the two samples should be positioned on the reference genome, which is the basis of subsequent data processing and analysis. Then there were many read segments captured by methylation sites in the IP samples, which would be mapped to the reference genome to form a reading segment enrichment region or a “peak” near the methylation sites. Therefore, the methylation enrichment point detection algorithm is called the peak calling algorithm. The m^6^A methylated peaks among the transcripts were identified by MACS (Zhang et al., 2008), and metagene m^6^A distribution was researched by MetaPlotR (Olarerin-George and Jaffrey, 2017). The DMGs were identified by diffReps (Shen et al., 2013). To explore the DMGs and DEGs from MeRIP-seq and RNA-seq, the Gene Ontology (GO) analysis and KEGG pathway enrichment analysis were performed.

### M^6^A-IP-qPCR and Reverse Transcription-qPCR

Ten genes with differentially methylated sites were verified by reverse transcription (RT)-qPCR according to m^6^A-seq. A small number of fragmented RNA was tested as the input control, while the rested RNA was incubated with anti-m^6^A antibody-coupled beads. Then the immunoprecipitation complex was eluted from the beads. In the end, RT-qPCR was performed on the input control and m^6^A -IP samples with gene-specific primers. Primers are listed in Supplementary Table S1.

### Cell Line Culture and Plasmid Transfection

In this study, RCC cell lines, including 786-O, ACHN, and OSRC, and human kidney proximal tubular epithelial cells HK-2 were used. These cell lines were purchased from the American Type Culture Collection (ATCC, Manassas, VA, United States) and National Infrastructure of Cell Line Resource, China. Cell lines were cultured in RPMI 1640 or DMEM with 10% fetal bovine serum (Invitrogen, Carlsbad, CA, United States) and incubated in a 5% CO_2_ environment at 37°C. The plasmids were transfected with Lipo3000 (Invitrogen) according to the protocol of the manufacturer, and the dosage of plasmids was 1 μg.

### Statistical Analysis

All statistical analyses were achieved and visualized using RStudio (version1.2.1335, Boston, MA, United States), GSEA (version4.0, UC San Diego and Broad Institute, United States) 23, Medcalc (version16.8, Ostend, Belgium), and GraphPad Prism (version 8.0, GraphPad, Inc., La Jolla, CA, United States). A two-tailed *p* < 0.05 was considered statistically significant.

## Results

### The m^6^A Content was Changed in Renal Cancer Cell

**FIGURE 1:**
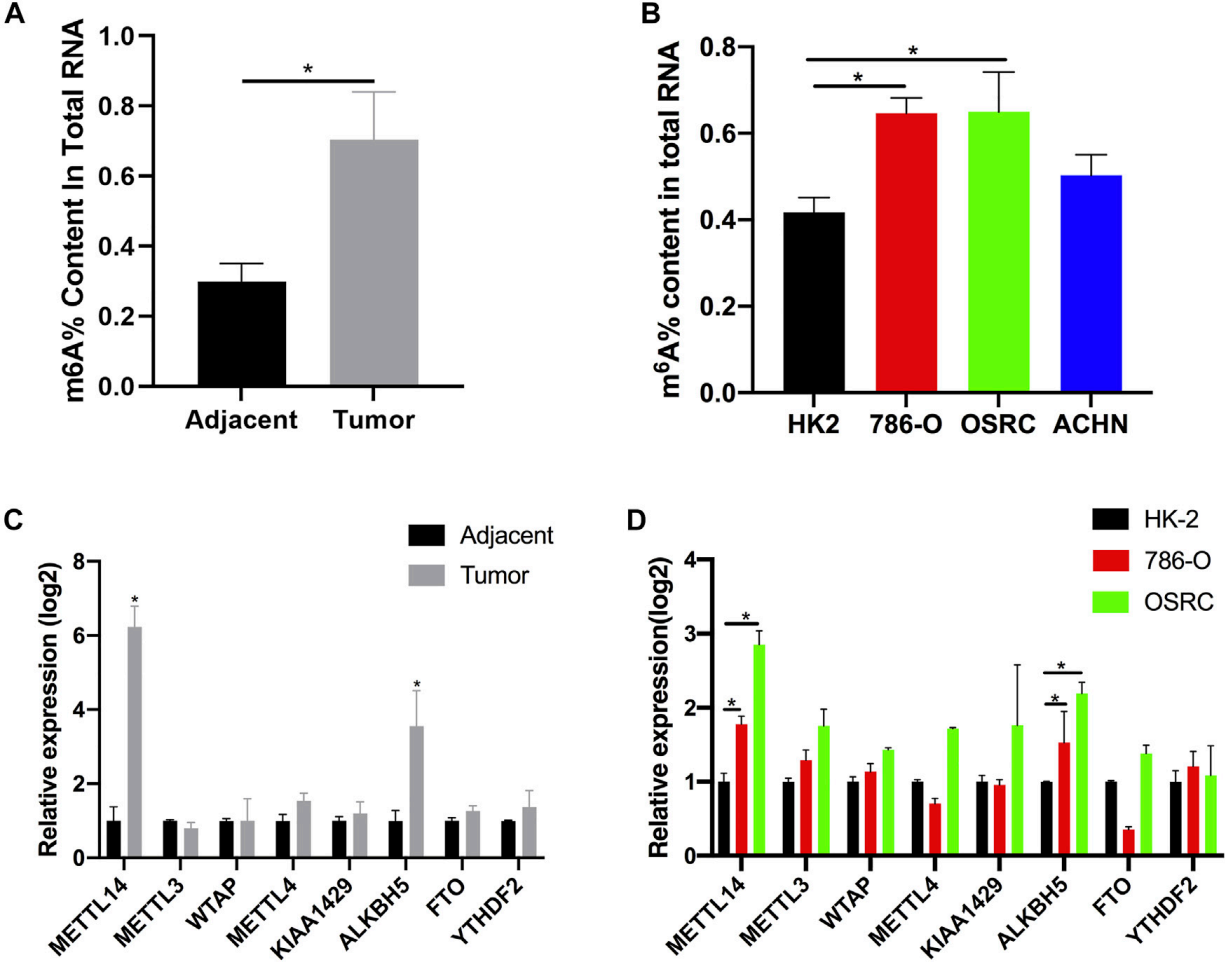
The N^6^-methyladenosine (m^6^A) contents and modification enzymes expression in renal cell carcinoma (RCC) tissues and RCC cells. **(A)** The m^6^A contents of total RNAs in tumor and adjacent tissues (*n* = 5). **(B)** The m^6^A contents of total RNAs in normal renal cell line (HK-2) and RCC cell lines (786-O, OSRC, and ACHN). **(C)** The mRNA expression levels of m^6^A modification enzymes in RCC and adjacent tissues (*n* = 5). **(D)** The mRNA expression levels of m^6^A modification enzymes in normal renal cell line (HK-2) and RCC cell lines (786-O and OSRC). *p < 0.05.

Recently, it has been found that RNA methylation could promote the progression of RCC (Dai et al., 2018). In order to explore the potential role of m^6^A modification in RCC, we measured the m^6^A content of total RNA in five pairs of tumor tissues and adjacent tissues using the m^6^A quantitative kit. We found the content was higher in tumor tissues than those in their corresponding normal tissues (Figure 1A). At the same time, we also examined the m^6^A contents of normal renal cell line HK-2 and renal cancer cell lines (786-O, OSRC, and ACHN), and found that m^6^A contents were also increased in RCC cells compared with normal cells (Figure 1B). m^6^A methyltransferase or demethylase could catalyze the modification of m^6^A. Therefore, we speculated that the abnormal m^6^A content might be caused by the abnormal expression of m^6^A methyltransferase or demethylase in RCC. In addition, many proteins have been found to be associated with m^6^A modification (Schwartz et al., 2014). To investigate our assumption, we examined the mRNA levels of eight genes associated with m^6^A modification in tissues and cell lines by RT-qPCR. Finally, the results showed that the mRNA expression levels of METL14 and ALKBH5 were increased in tumor tissues and RCC cell lines, while the mRNA expression levels of other genes were not significantly changed (Figures 1C,D).

### The mRNA m^6^A Modification was Dynamic and Differed Between Paired Renal Tumor Tissues and Adjacent Tissues

**FIGURE 2:**
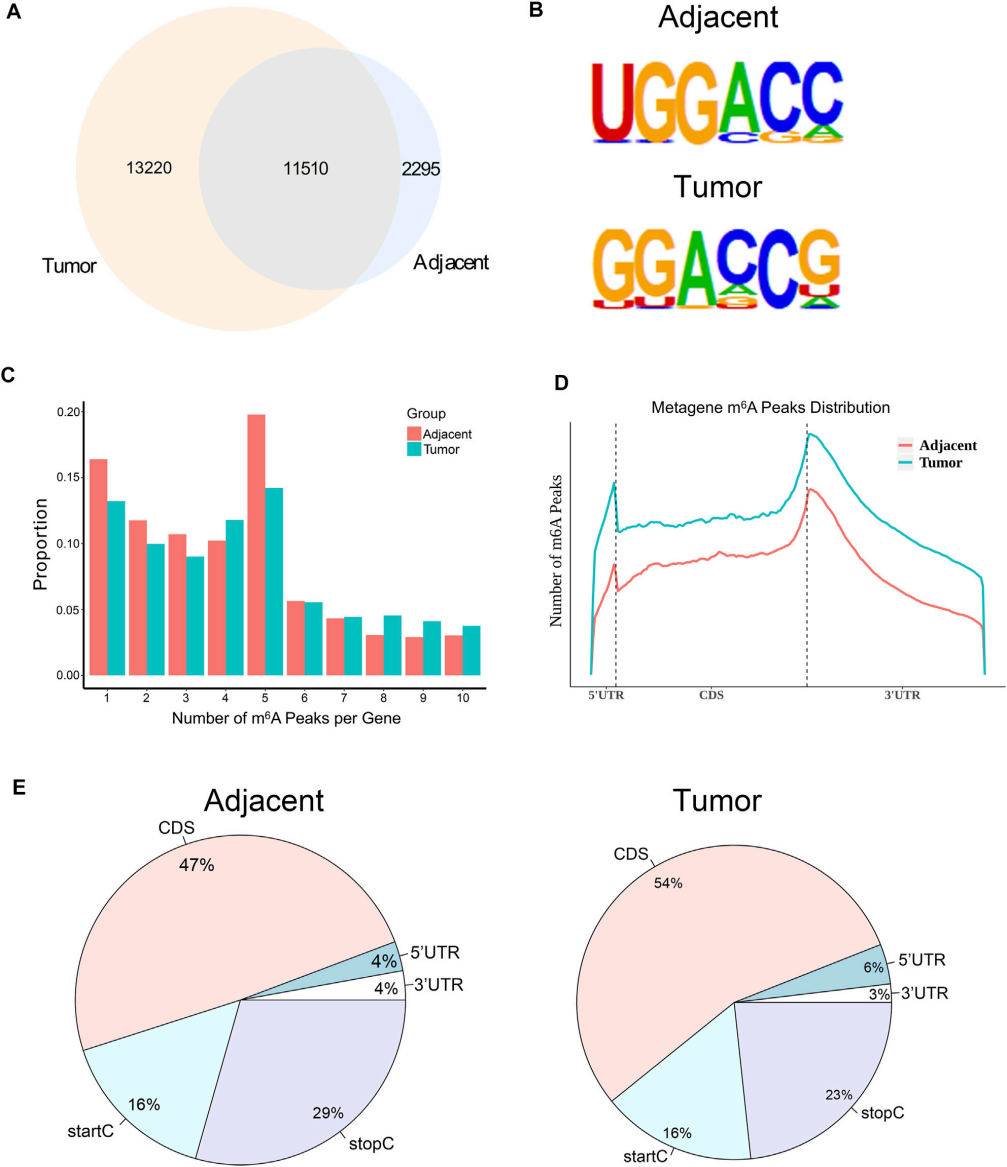
The mRNA m^6^A profile of five paired renal tumor tissues and adjacent tissues. **(A)** The Venn diagram showing the numbers of m^6^A peaks in tumor and adjacent tissues. **(B)** The sequence motif of m^6^A-containing peak regions in tumor and adjacent tissues, respectively. **(C)** The distribution of m^6^A modifications in the human transcriptome. **(D)** The metagene profiles of transcripts peaks in RCC tissues and adjacent tissues. **(E)** The proportion of m^6^A peaks located at transcripts. startC, start codon; stopC, stop codon.

In order to investigate whether m^6^A modification could promote the progression of RCC, we used MeRIP-seq (Dominissini et al., 2013) to detect the differences of m^6^A level in the paired renal tumor tissues and adjacent tissues. MeRIP-seq analysis revealed that there were 13,805 m^6^A peaks among 5,568 coding gene transcripts (mRNAs) in adjacent tissues, and 24,730 m^6^A peaks among 6,866 mRNAs in tumor tissues. Of these, 11,510 peaks were overlapped between the adjacent tissues and tumor tissues (Figure 2A). The low overlapping m^6^A peaks of mRNAs suggested that there were differences in the m^6^A patterns between the two groups. To study whether the m^6^A peaks had a conserved motif (Wei et al., 1976; Schibler et al., 1977), the m^6^A peaks identified from the MeRIP-seq were analyzed by the HOMER motif software (Heinz et al., 2010). The results showed that there was a difference of m^6^A motif between tumor tissues and adjacent tissues, while their motifs were similar to those previously reported (Fu et al., 2014; Chen et al., 2015) (Figure 2B). Besides, we examined the distribution of m^6^A modifications in the human transcriptome. We found that 70% of methylated sequences in the adjacent group (60% of methylated transcripts in the tumor group) contained fewer than five m^6^A sites, while fewer transcripts contained six or more sites (Figure 2C).

In order to determine the priority position of m^6^A in transcripts, we then studied the metagene profiles of the peaks in RCC tissues and adjacent normal tissues. We found that most m^6^A peaks were located at the end of the 5′UTRs and start of the 3′UTRs (Figure 2D). At the same time, we found that the proportion of m^6^A peaks in CDS was the highest and that in UTRs was the lowest in both tissues (Figure 2E). These results of the m^6^A modification distributions were similar to those reported previously (Dominissini et al., 2012; Meyer et al., 2012).

### mRNA Containing Differential m^6^A Sites was Enriched in Kidney Development and Cancer-Related Signaling Pathways

**FIGURE 3:**
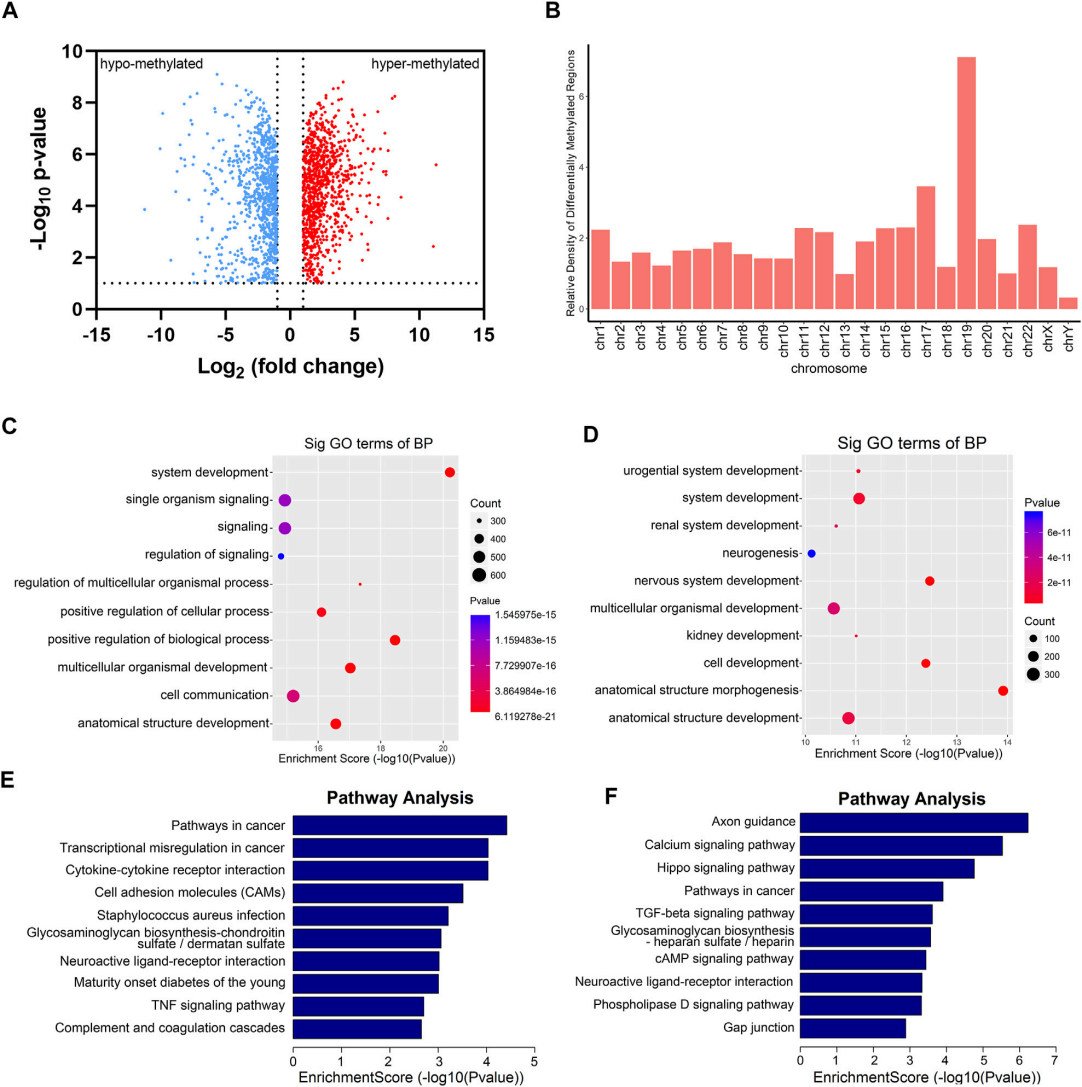
Gene Ontology (GO) and KEGG analysis of DMGs between five paired renal tumor and adjacent tissues. **(A)** Volcano blot of differentially methylated mRNAs (DMGs) in tumor compared with adjacent tissues. **(B)** The container of differentially methylated m6A sites harbored by different chromosomes. **(C)** GO analysis of hypermethylated genes in RCC tumor tissues. **(D)** GO analysis of hypomethylated genes in RCC tumor tissues. **(E)** KEGG analysis of hypermethylated genes in RCC tumor tissues. **(F)** KEGG analysis of hypomethylated genes in RCC tumor tissues.

Totally, we had identified 4,404 differential m^6^A sites (DMMSs) within 1,877 nuclear mRNAs, of which 43% (1,887*/*4,404) were significantly downmethylated sites (tumor vs. adjacent). Compared with the adjacent group, we found 923 significantly hypomethylated coding genes and 954 significantly hypermethylated coding genes in the tumor group (Figure 3A). Besides, we analyzed the relative density of differential m^6^A modification sites on chromosomes; the top three chromosomes were 19, 17, and 22 (Figure 3B). To explore the functions of m^6^A in RCC, differentially methylated genes were performed for Gene Ontology enrichment analysis and Kyoto Encyclopedia of Genes and Genomes pathway analysis. The results indicated that differentially methylated genes in the tumor group were mostly enriched in many system development-associated processes (Figures 3C,D). Furthermore, differentially methylated genes were found to be significantly involved in cancer pathways, such as the transcriptional misregulation and TGF-β signaling pathway (Figures 3E,F).

### Differentially Expressed RNAs were Involved in Important Biological Pathways

**FIGURE 4:**
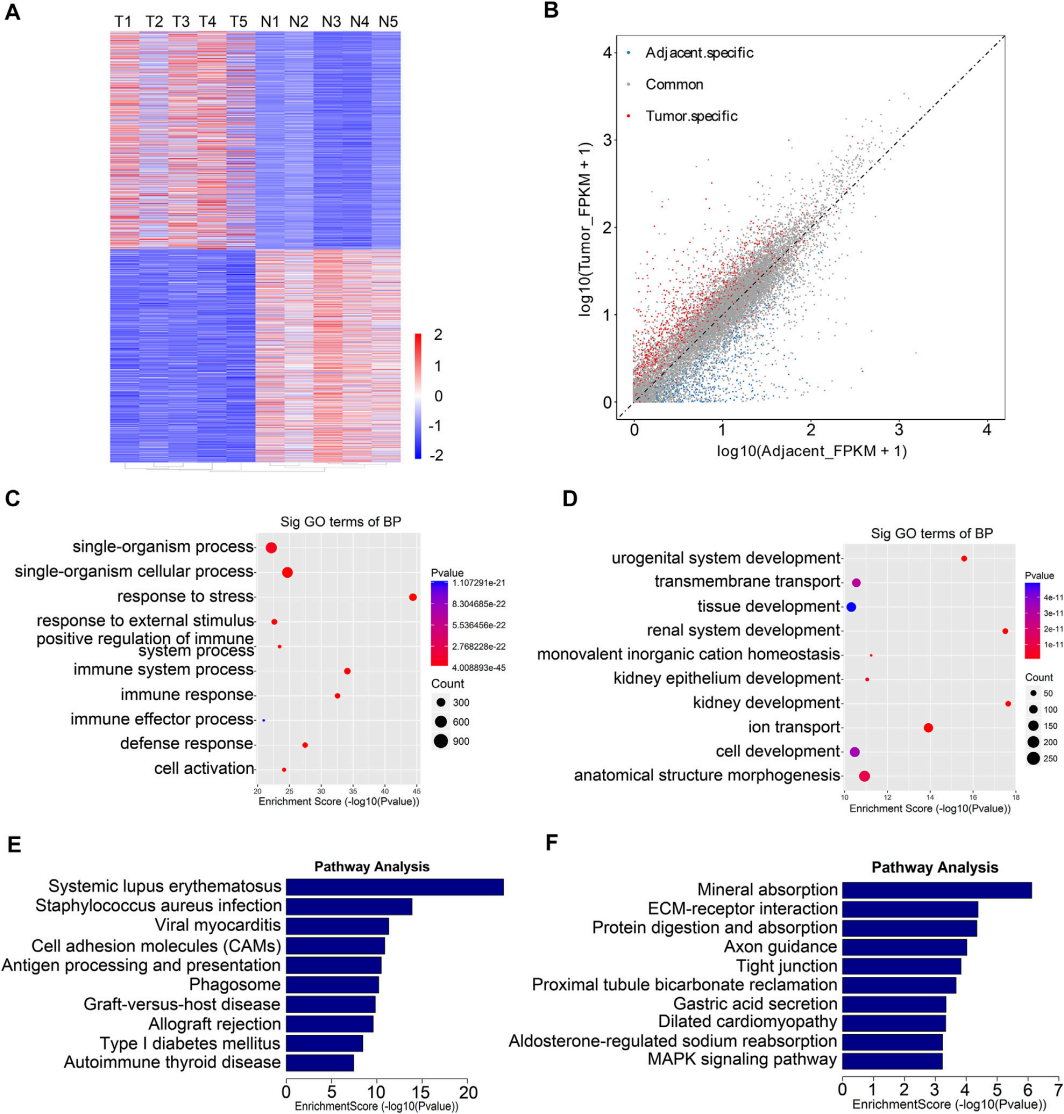
Functional analysis of differentially expressed genes between five paired renal tumor and adjacent tissues. **(A)** Heatmap of differentially expressed mRNAs in renal tumor tissues and adjacent tissues (T, tumor group; N, adjacent normal group). **(B)** Scatter plots showing the tissue specific DEGs (fold changes ≥2 and *p* < 0.05). **(C)** GO analysis of upregulated genes in RCC tumor tissues. **(D)** GO analysis of downregulated genes in RCC tumor tissues. **(E)** KEGG analysis of upregulated genes in RCC tumor tissues. **(F)** KEGG analysis of downregulated genes in RCC tumor tissues.

From the RNA-seq data, we found that 1,469 genes were upregulated in tumor tissues, and 1,402 were upregulated in adjacent tissues (Figures 4A,B). Next, we performed Gene Ontology enrichment analysis and Kyoto Encyclopedia of Genes and Genomes pathway analysis to investigate the function of the differentially expressed RNAs. We found that the differentially expressed RNAs were mainly related to single-organism process and urogenital system development (Figures 4C,D). Moreover, pathway analysis showed that systemic lupus erythematosus, tight junction, and MAPK signaling pathway were significantly changed in tumor samples (Figures 4E,F).

### Conjoint Analysis of MeRIP-Seq and RNA-Seq

**FIGURE 5:**
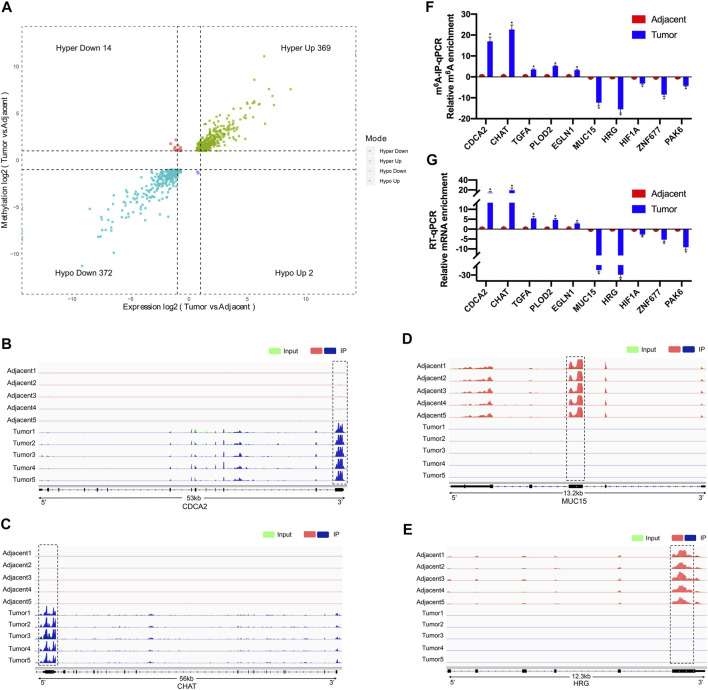
Conjoint analysis of differentially methylated genes and differentially expressed genes between five paired renal tumor and adjacent tissues. **(A)** Four quadrant diagram showing the differentially methylated genes and differentially expressed genes. **(B,C)** Integrative Genome Viewer (IGV) software showing the m^6^A peaks in hypermethylated and upregulated gene cell division cycle-associated 2 (CDCA2) **(B)** and choline-O-acetyltransferase (CHAT) **(C)**. **(D,E)** Integrative Genome Viewer (IGV) software showed the m^6^A peaks in hypomethylated and downregulated genes mucin 15 (MUC15) **(D)** and histidine-rich glycoprotein (HRG) **(E)**. **(F)** Validations of the m^6^A enrichment of five hypermethylated genes (CDCA2, CHAT, TGFA, PLOD2, and EGLN1) and five hypomethylated genes (MUC15, HRG, HIF1A, ZNF677, and PAK6) by m^6^A-immunoprecipitation (IP)-qPCR (*n* = 5). **(G)** Validations of the mRNA expression level of five upregulated genes (CDCA2, CHAT, TGFA, PLOD2, and EGLN1) and five downregulated genes (MUC15, HRG, HIF1A, ZNF677, and PAK6) by RT-qPCR (*n* = 5). *p < 0.05.

After conjoint analysis of meRIP-seq and RNA-seq, 369 hyper-up genes, 372 hypo-down genes, 14 hyper-down genes, and two hypo-up genes were found in RCC tissues compared with adjacent tissues (Figure 5A). This result promoted us to investigate the cancer-related genes in the RCC. For instance, cell division cycle-associated 2 (CDCA2) promotes the proliferation and development of colon cancer, within which m^6^A was hypermethylated (tumor vs. adjacent) near the stop codon (Figure 5B) (Feng et al., 2019), and the m^6^A peaks of choline-O-acetyltransferase (CHAT) (Yokomori et al., 1983; Pahud et al., 2001) were enriched around the 5′UTR of CHAT in tumors (Figure 5C), while mucin 15 (MUC15), a significantly hypermethylated peak enriched in coding sequence (CDS) was shown in the adjacent groups (Figure 5D), which had been reported to promote the progression of cancer (Zhang et al., 2020). It was reported that HRG could prevent the cancer development by inducing macrophage polarization (Rolny et al., 2011), and its m^6^A peak was enriched around the beginning of the 3′UTR in the adjacent groups in our study (Figure 5E).

To further confirm the results of our m^6^A-seq data, we analyzed gene-specific m^6^A-IP qPCR assays for several hypermethylated genes (CDCA2, CHAT, TGFA, PLOD2, and EGLN1) and hypomethylated genes (MUC15, HRG, HIF1A, ZNF677, and PAK6), which might participate in RCC development. We verified the same m^6^A level changes of 10 genes, confirming the validity of the meRIP-seq results (Figure 5F). Next, mRNA levels of the abovementioned 10 genes were measured in the five pairs of adjacent and tumor samples by RT-qPCR (Figure 5G). Results showed a similar tendency of m^6^A-methylated levels and mRNA expressions in the two groups, which suggested a relationship between m^6^A mRNA methylation and gene transcription.

### dCas13b-ALKBH5 Fusion Protein Induced Demethylation of m^6^A-Modified mRNAs *in vitro*

**FIGURE 6:**
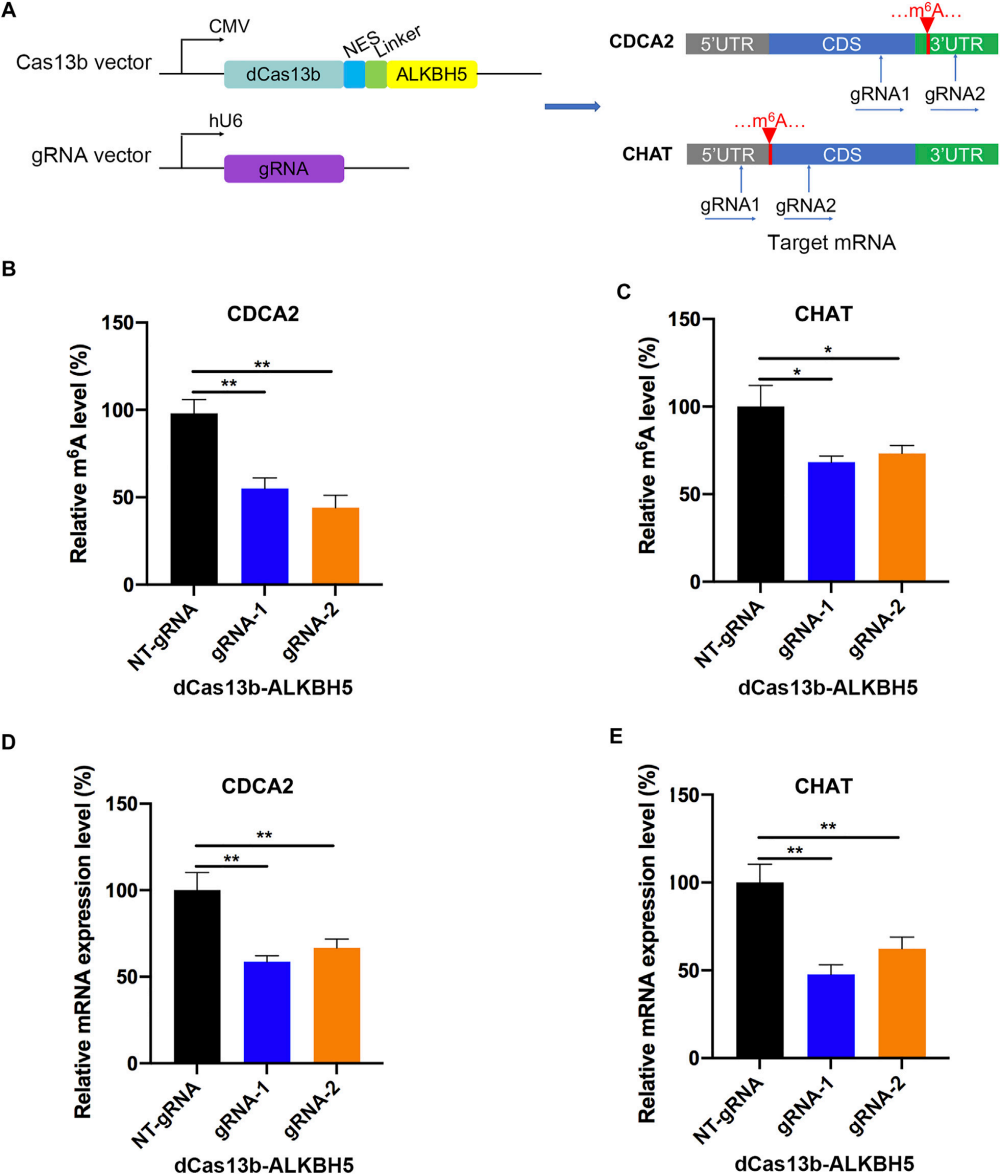
dCas13b-ALKBH5 induces demethylation of m^6^A hypermethylated mRNA in RCC cells. **(A)** The design of dCas13b-ALKBH5 fusion protein and sgRNAs targeted with CDCA2 and CHAT mRNA m^6^A sites. **(B,C)** The m^6^A level of CDCA2 **(B)** and CHAT **(C)** mRNA in RCC cell line 786-O after transfected with dCas13b-ALKBH5 and gRNAs. **(D**,**E)** The mRNA levels of CDCA2 **(D)** and CHAT **(E)** in 786-O cell after transfection with dCas13b-ALKBH5 and gRNAs. **p < 0.01.

To study mRNA modifications *in vitro*, we performed dCas13b-ALKBH5 fusion protein for targeted mRNA demethylation (Li et al., 2020a). We designed two guide RNAs targeting the 3′UTR or CDS of CDCA2 mRNA, and another two gRNAs targeting 5′UTR or CDS of CHAT mRNA. These two mRNAs were hypermethylated in RCC, then all four gRNAs were transfected in 786-O RCC cell line with dCas13b-ALKBH5 fusion protein, respectively (Figure 6A). The results of m^6^A-IP qPCR revealed that the m^6^A levels of the targeted site were decreased after transfecting with dCas13b-ALKBH5 and gRNAs, compared with NT-gRNA (Figures 6B,C). Intriguingly, the mRNA expression levels of the targeted CDCA2 and CHAT were decreased after transfecting gRNAs (Figures 6D,E). YTHDF1, which is responsible for binding to the translational machinery and RNA translation activation, might be inhibited by dCas13b-ALKBH5 (Wang et al., 2015). Collectively, these results suggested that dCas13b-ALKBH5 could demethylate m^6^A levels of targeted hypermethylated mRNAs.

### dCas13b-M3M14 Fusion Protein Induced Methylation of m^6^A-Modified mRNAs *in vitro*

**FIGURE 7:**
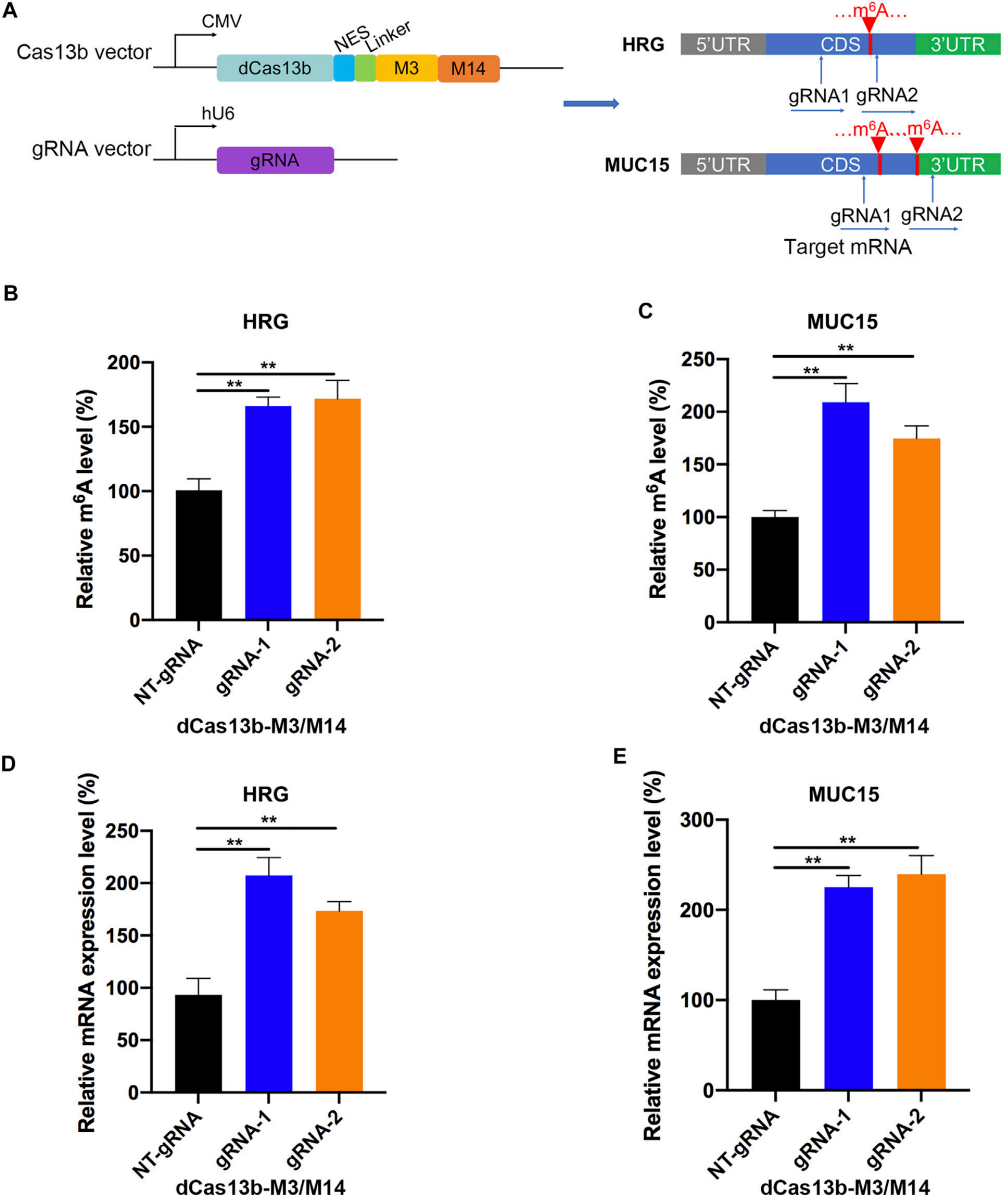
dCas13b-M3M14 induces methylation of m^6^A hypomethylated mRNA in RCC cells. **(A)** The design of dCas13b-M3M14 fusion protein and sgRNAs targeted with MUC15 and HRG mRNA m^6^A sites. **(B,C)** The m^6^A level of MUC15 **(B)** and HRG **(C)** mRNA in RCC cell line 786-O after transfection with dCas13b-ALKBH5 and gRNAs. **(D,E)** The mRNA levels of MUC15 **(D)** and HRG **(E)** in 786-O cell after transfection with dCas13b-ALKBH5 and gRNAs. **p < 0.01.

In addition, we constructed the dCas13b-M3M14 fusion protein (Wilson et al., 2020) to promote m^6^A modifications in RCC cells. The mRNAs of HRG and MUC15 were hypomethylated in RCC and were targeted by two gRNAs at different positions (Figure 7A). First, we measured the m^6^A levels of HRG and MUC15 in 786-O RCC cell lines transfected with dCas13b-M3M14 and gRNAs or NT-gRNAs. The results showed that dCas13b-M3M14 significantly increased the m^6^A levels of HRG and MUC15, suggesting that gRNAs could efficiently recognize their targeted mRNAs (Figures 7B,C). We then verified the effect of dCas13b-M3M14 fusion protein (gRNAs for HRG and MUC15) on the mRNA expressions of HRG and MUC15. The results of RT-qPCR showed that dCas13b-M3M14 fusion protein transfected with gRNAs significantly upregulated the mRNA levels of their targeted mRNAs (Figures 7D,E). This might be due to dCas13b-M3M14 fusion protein increasing the binding of targeted mRNA and YTHDF1. Finally, this dCas13b-M3M14 fusion protein could mediate efficient installation of m^6^A in endogenous RNA transcripts.

## Discussion

m^6^A is identified as a dynamic and reversible RNA modification in eukaryotes, due to the “writer” (methyltransferase) and “eraser” (demethylase) proteins. It has been reported that m^6^A modification could take part in many cellular activities and reaction, including heat shock (Zhou et al., 2015), ultraviolet light (Robinson et al., 2019), hypoxic stress (Fry et al., 2017), and oxidative stress (Anders et al., 2018). Moreover, there were numbers of evidences confirming that m^6^A modification could promote the development of tumors. In this study, we identified many differentially methylated genes in RCC samples and tumor-adjacent normal tissues based on meRIP-seq technology, analyzed and validated gene expression and cancer-related pathways modulated by abnormal m^6^A RNA modifications.

We figured out that m^6^A modification in tumor tissues and normal tissues mainly occurred in the GGACC motif, which was similar to the previous data, and the m^6^A peaks of transcripts were mainly located at the CDS site. Almost 80% of the methylated genes had one to five m^6^A sites, and others contained over eight m^6^A sites in mRNAs. In the current study, differentially methylated mRNAs between tumor and normal tissues were shown to be involved in many important biological pathways. As observed, the gene function analyses of DMMSs showed that the hyper- and hypomethylated genes in the tumor group were significantly enriched in many phylogenetic processes, such as multicellular organismal development and kidney development, and they were also involved in the cancer pathways, such as the transcriptional misregulation in cancer and TGF-β signaling pathway, which supported the importance of m^6^A in tumorigenesis. It was reported that m^6^A could play an important role in carcinogenesis and the development of gastric cancer, and the genes with higher m6A levels were mainly enriched in transcriptional misregulation in carcinogenesis pathways, whereas the genes with decreased methylation mainly regulate digestion and absorption of protein (Sang et al., 2020). In addition, the dysregulated expression of m^6^A was also involved in transcriptional misregulation in cancer and other malignancy-related pathways including primary immunodeficiency, regulation of autophagy, and response to oxidative stress (Zheng et al., 2020). It was also reported that the expression of m^6^A methyltransferase METTL3 and m^6^A modification were increased during TGF-β-induced epithelial–mesenchymal transition in A549 and LC2/ad lung cancer cells, and mechanistic investigations revealing that METTL3 could be indispensable for TGF-β-induced epithelial–mesenchymal transition of lung cancer cells through the regulation of JUNB (Wanna-Udom et al., 2020). Moreover, m^6^A modification was involved in the epithelial–mesenchymal transition of cancer cells by regulating the expression and secretion of TGF-β1 (Li et al., 2020b). These studies revealed that differential m^6^A modifications were involved in important biological pathways, which were consistent with our study.

Combined analysis of m^6^A-seq and mRNA-seq data uncovered 369 hyper-up genes and 372 hypo-down genes in tumor tissues compared with adjacent normal tissues, which might play critical roles in the RCC development. Moreover, some of the genes have been reported to facilitate tumor growth and metastasis in different types of cancers. To further investigate mRNA m^6^A modification of these specific genes, we used CRISPR-dCas13b fusion proteins to regulate the methylation levels of mRNA. For instance, the methylation levels of CDCA2 and CHAT were found to be approximately 20 times higher in the tumor group than that in the control group. Then, we applied dCas13b-ALKBH5 fusion protein to induce the demethylation of CDCA2 and CHAT mRNAs in RCC cell lines, and found that the m^6^A levels and mRNA expression levels were significantly reduced after transfecting with their targeted gRNAs. Furthermore, another two hypomethylated genes, MUC15 and HRG, exhibited downregulated mRNA levels in the tumor group. Therefore, we performed dCas13b-M3M14 fusion protein combined with two gRNAs to methylate mRNA m^6^A level of MUC15 and HRG in RCC cell line. The data showed hypermethylated m^6^A levels and upregulated mRNA levels of MUC15 and HRG, which were in line with our expectations.

However, the study was still partially flawed. We cannot avoid the potential selection bias, since the m^6^A-RIP-seq and RNA-seq were based on five paired ccRCC tumor tissues and adjacent tissues. Further functional experiments and mechanism explorations of m^6^A modification in ccRCC should be performed. Despite the defects listed above, the presented findings still provided a link between mRNA m^6^A modifications and renal tumorigenesis, which could be expected to be a new target for gene targeted therapy of renal cell carcinoma.

## Data Availability

The original contributions presented in the study are publicly available. This data can be found here: https://www.ncbi.nlm.nih.gov/bioproject/PRJNA719065.
