# Supplementary figures and images for: m6A-mRNA Methylation Regulates Gene Expression and Programmable m6A Modification of Cellular RNAs With CRISPR-Cas13b in Renal Cell Carcinoma
Source: Front Genet. 2022 Jan 21;12:795611. doi: 10.3389/fgene.2021.795611 (PMC8815861; doi:10.3389/fgene.2021.795611)

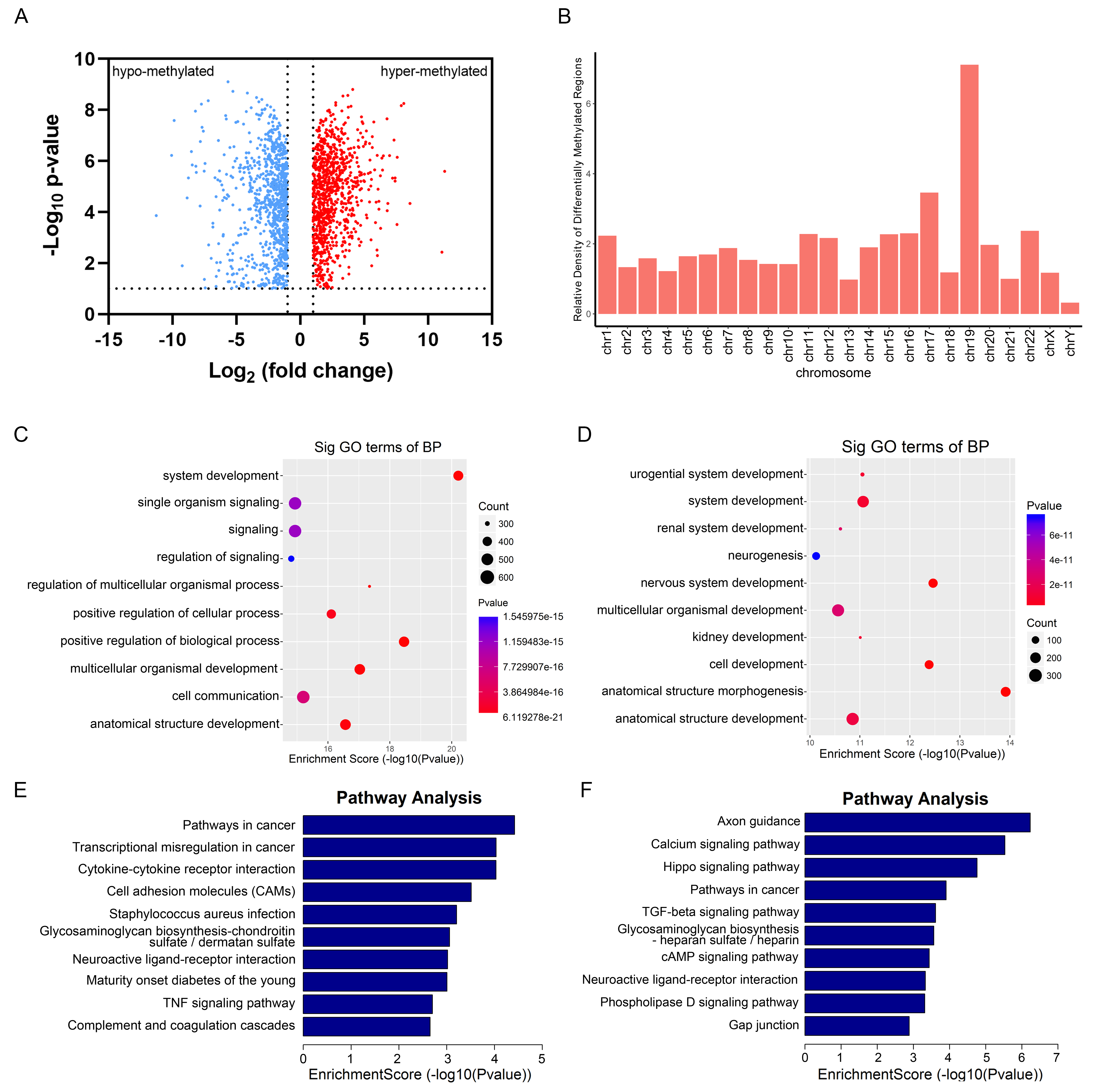

Supplement: Supplementary file 1 [file DataSheet1.ZIP › F-3.tif]

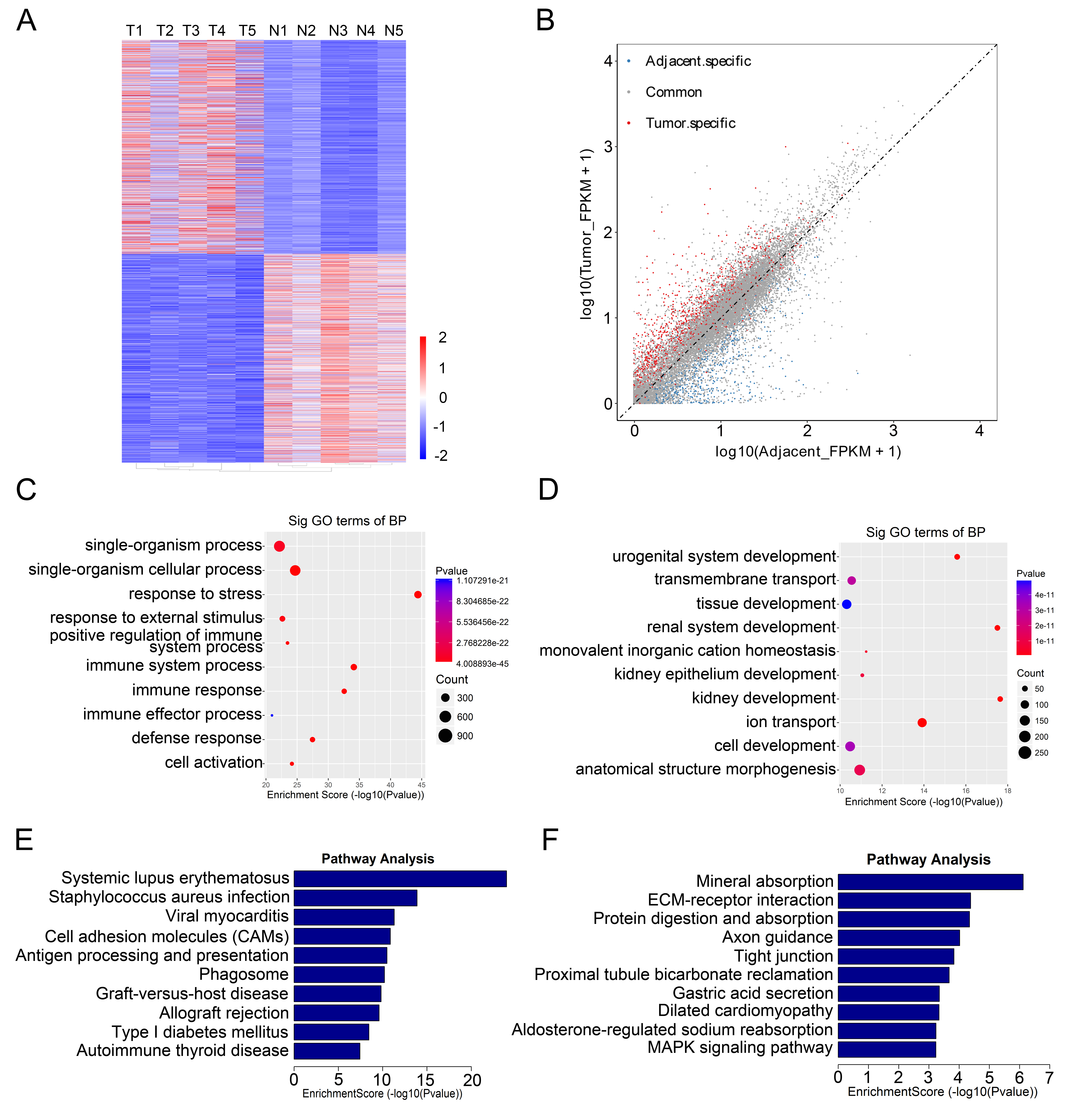

Supplement: Supplementary file 1 [file DataSheet1.ZIP › F-4.tif]

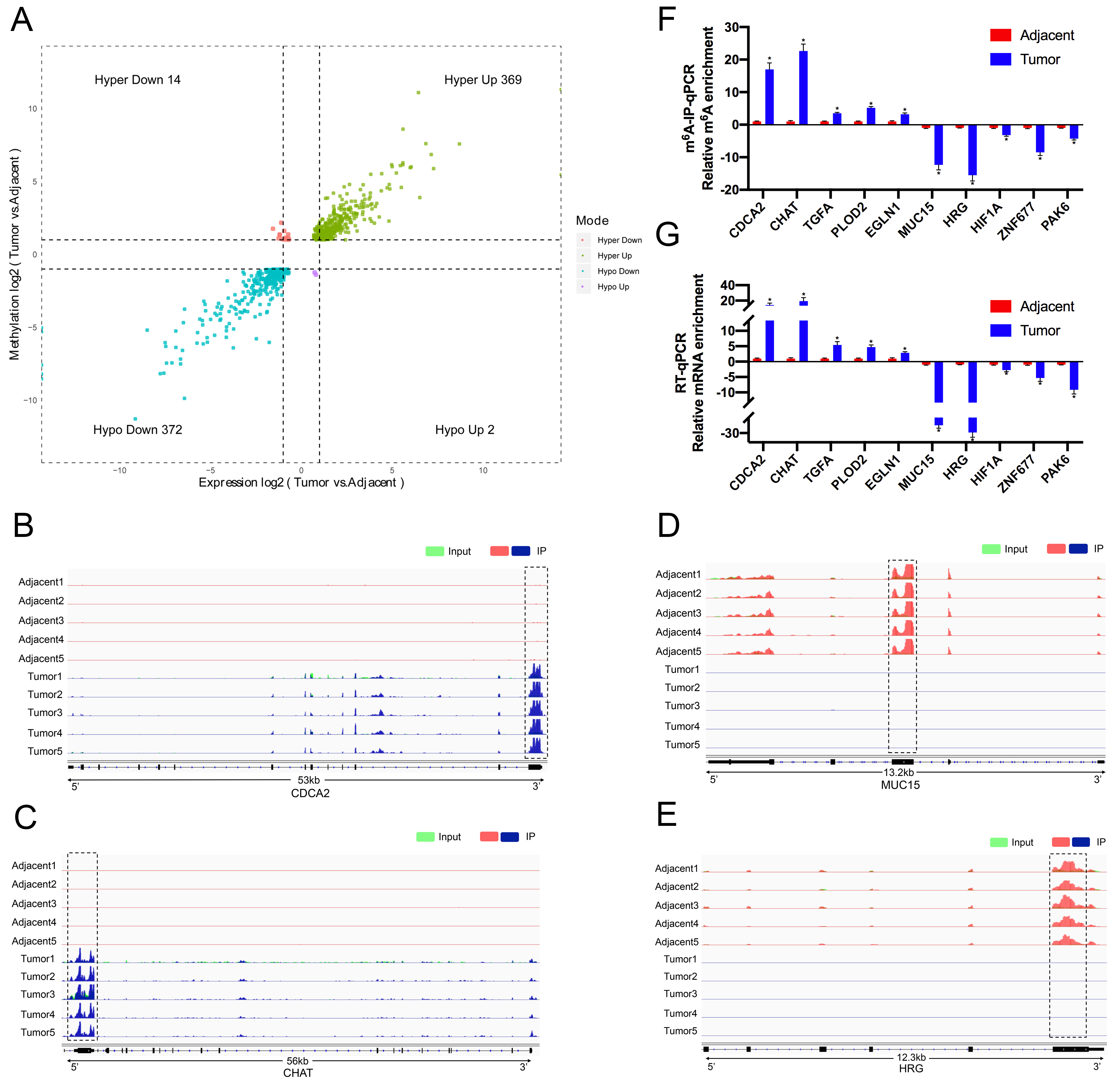

Supplement: Supplementary file 1 [file DataSheet1.ZIP › F-5.tif]

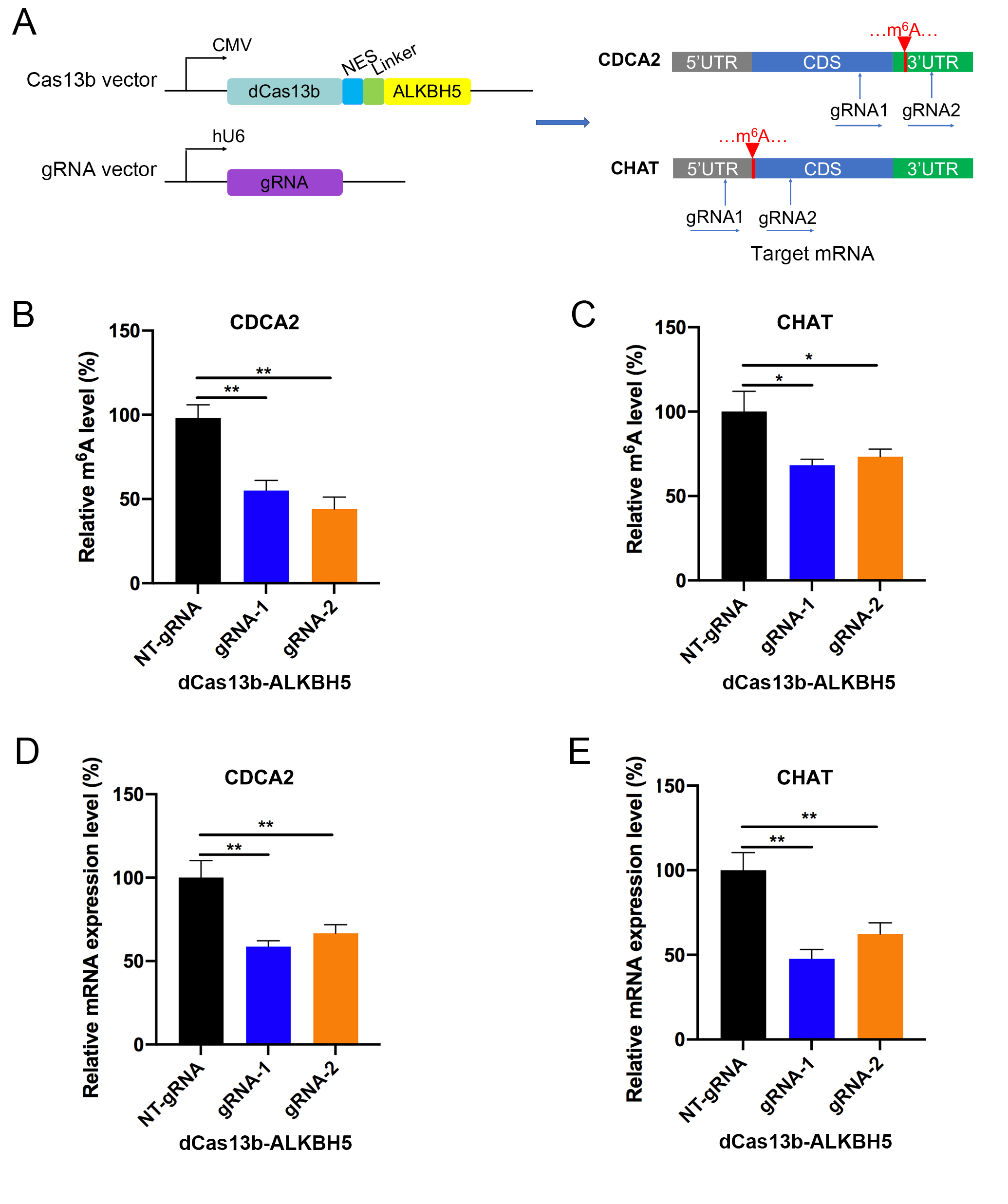

Supplement: Supplementary file 1 [file DataSheet1.ZIP › F-6.tif]

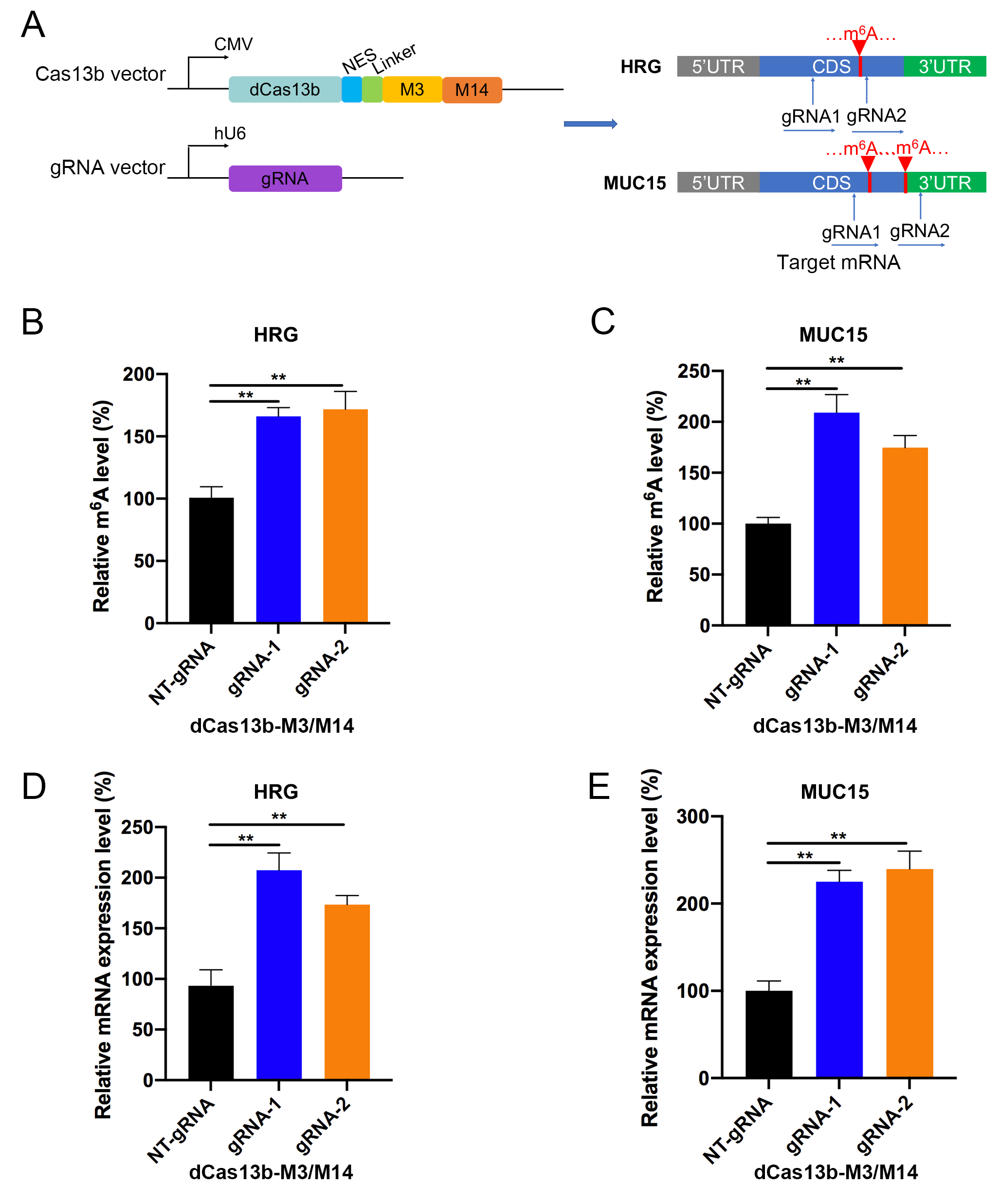

Supplement: Supplementary file 1 [file DataSheet1.ZIP › F-7.tif]

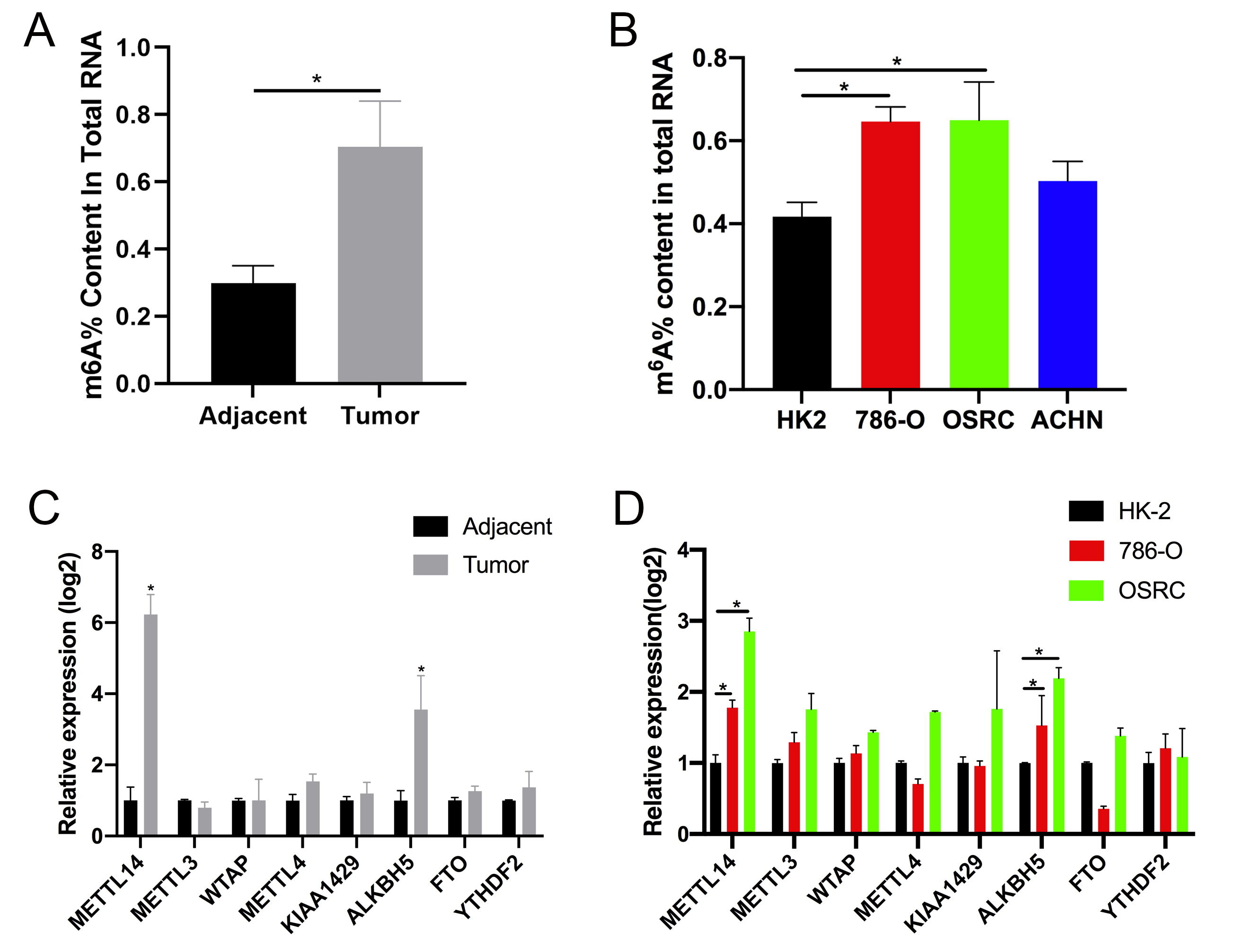

Supplement: Supplementary file 1 [file DataSheet1.ZIP › F-1.tif]

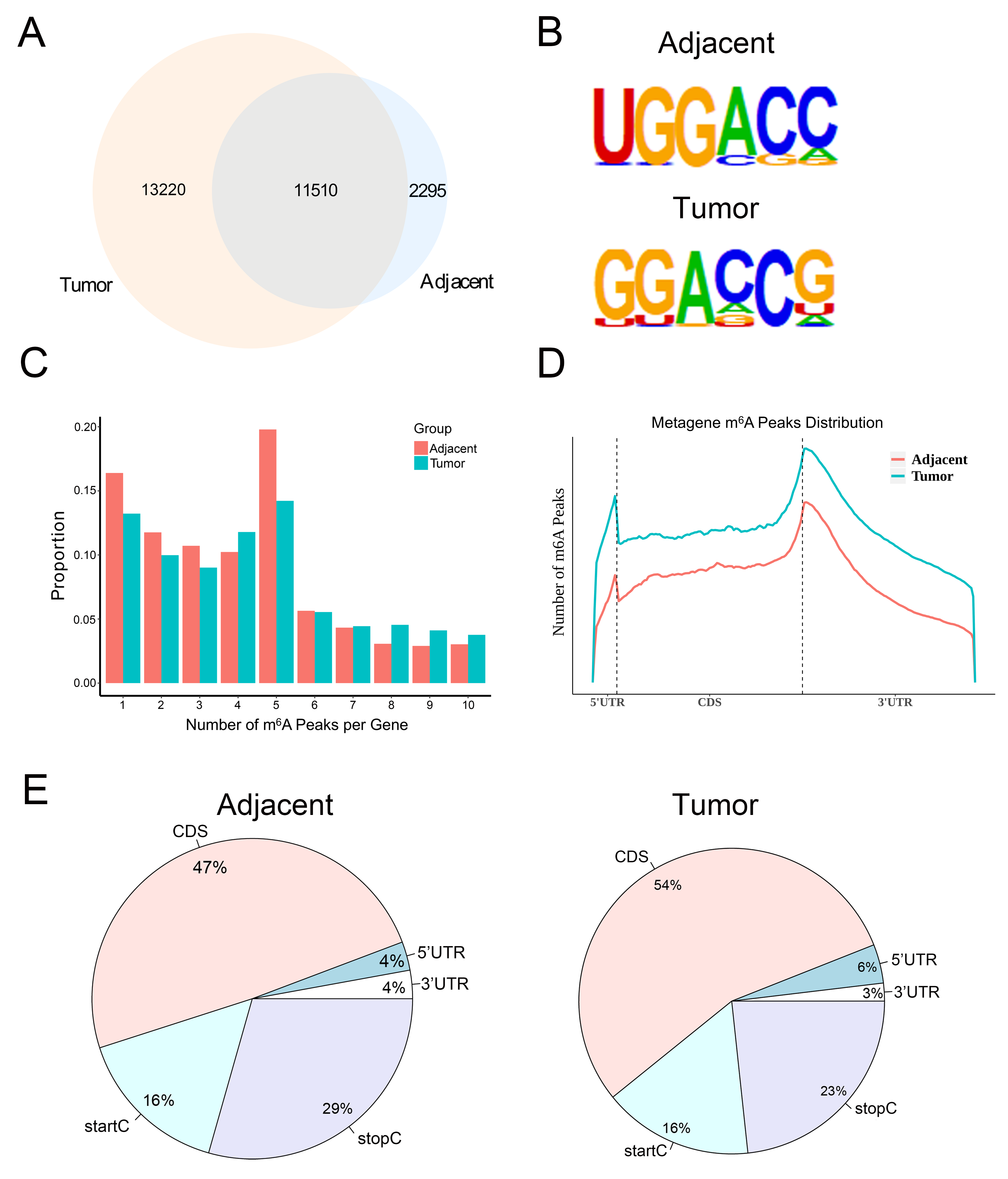

Supplement: Supplementary file 1 [file DataSheet1.ZIP › F-2.tif]
