# Supplementary material for: m6A-mRNA Methylation Regulates Gene Expression and Programmable m6A Modification of Cellular RNAs With CRISPR-Cas13b in Renal Cell Carcinoma
Source: Front Genet. 2022 Jan 21;12:795611. doi: 10.3389/fgene.2021.795611 (PMC8815861; doi:10.3389/fgene.2021.795611)
Supplement: Supplementary file 2 [file DataSheet2.docx]

| Target | Sequence (5'-3') | Application |
| --- | --- | --- |
| hGAPDH-qPCR-F | CGGATTTGGTCGTATTGGG | RT-qPCR |
| hGAPDH-qPCR-R | CTGGAAGATGGTGATGGGATT |  |
| hMETTL14-qPCR-F | AGAAACTTGCAGGGCTTCCT |  |
| hMETTL14-qPCR-R | TCTTCTTCATATGGCAAATTTTCTT |  |
| hMETTL3-qPCR-F | AAGCTGCACTTCAGACGAAT |  |
| hMETTL3-qPCR-R | GGAATCACCTCCGACACTC |  |
| hWTAP-qPCR-F | GGCGAAGTGTCGAATGCT |  |
| hWTAP-qPCR-R | CCAACTGCTGGCGTGTCT |  |
| hMETTL4-qPCR-F | TAAAGACGGCAAGACCAT |  |
| hMETTL4-qPCR-R | TGATGCCAGGAAAGAACA |  |
| hKIAA1429-qPCR-F | GAATACTGATGGTCTGGTGCTA |  |
| hKIAA1429-qPCR-R | CTTGGCTGTGGTCTTGGA |  |
| hFTO-qPCR-F | TGGGTTCATCCTACAACGG |  |
| hFTO-qPCR-R | CCTCTTCAGGGCCTTCAC |  |
| hALKBH5-qPCR-F | CCCGAGGGCTTCGTCAACA |  |
| hALKBH5-qPCR-R | CGACACCCGAATAGGCTTGA |  |
| hYTHDF2-qPCR-F | GGCAGCACTGAAGTTGGG |  |
| hYTHDF2-qPCR-R | CTATTGGAAGCCACGATGTTA |  |
| CDCA2-F | CAGCCCTGCACTGTATCGAA |  |
| CDCA2-R | ACAGCCGGTCATTTTCTCGT |  |
| CHAT-F | GGTTTCACCTCCCAAACCCA |  |
| CHAT-R | GGTCTGTGAGTTGGACCCTG |  |
| CHAT-F | GTTTGCCTCCAATTGGCCTG | meRIP |
| CHAT-R | GTTTGCCTCCAATTGGCCTG |  |
| EGLN1-F | CATCTGTGTGGTGGACGACT |  |
| EGLN1-R | CTCGGATGTCCTTGGACGAG |  |
| EGLN1-F | CGCACAGGCCCTATTCTCTC | meRIP |
| EGLN1-R | CGCACAGGCCCTATTCTCTC |  |

**Table 1 The sequence of Primers in RT-qPCR and meRIP**
